# Impact of changes in head position during head and neck surgery on the depth of tracheal tube intubation in anesthetized children

**DOI:** 10.1186/s12871-020-01033-7

**Published:** 2020-05-24

**Authors:** Siyi Yan, Huan Zhang

**Affiliations:** Department of Anesthesiology, Beijing Tsinghua Changgung Hospital, No.168, LiTang Road, ChangPing District, Beijing, China

**Keywords:** Head and neck surgery, Depth of Oral trachea cannula, Position changes, Tracheal tube intubation, Children

## Abstract

**Background:**

The classic formula has been used to estimate the depth of tracheal tube intubation in children for decades. However, it is unclear whether this formula is applicable when the head and neck position changes intraoperatively.

**Methods:**

We prospectively reviewed the data of 172 well-developed children aged 2–12 years (64.0% boys) who underwent head and neck surgery under general anesthesia. The distances from the tracheal carina to the endotracheal tube tip (CT), from the superior margin of the endotracheal tube tip to the vocal cord posterior commissure (CV), and from the tracheal carina to the posterior vocal commissure (TV) were measured in the sniffing position (maximum), neutral head, and maximal head flexion positions.

**Results:**

Average CT and CV in the neutral head position were 4.33 cm and 10.4 cm, respectively. They increased to 5.43 cm and 11.3 cm, respectively, in the sniffing position, and to 3.39 cm and 9.59 cm, respectively, in the maximal flexion position (all *P*-values < 0.001). TV remained unchanged and was only dependent on age. After stratifying patients by age, similar results were observed with other distances. CT and CV increased by 1.099 cm and 0.909 cm, respectively, when head position changed from neutral head to sniffing position, and decreased by 0.947 cm and 0.838 cm, respectively, when head position changed from neutral head to maximal flexion.

**Conclusion:**

Change in head position can influence the depth of tracheal tube intubation. Therefore, the estimated depth should be corrected according to the surgical head position.

## Background

Inappropriate placement of tracheal tube can lead to incidences of perioperative respiratory complications in pediatric patients [1, 2]. If the tracheal tube is placed too shallow, the catheter cuff is directly clamped onto the vocal cords, causing air leakage during mechanical ventilation, leakage of oropharynx secretions, and entry of blood from the surgical field into the airway, which results in aspiration pneumonia or vocal cord damage, and even hoarseness. In contrast, if the tracheal tube is placed too deep, it might damage the tracheal carina or cause endobronchial intubation, possibly resulting in single lung ventilation, hypoxemia, and finally, lung damage.

There are several simple formulas to calculate the depth of orotracheal intubation in children over 1 year of age, which are mainly based on body weight, body length, and age. All these formulas have been widely used in clinical practice for many decades. However, a recent meta-analysis of 16 published studies found that only 81% of catheter placements after orotracheal cannulation were appropriate when using the advanced pediatric life support (APLS) [3], and that the rate of catheter tip malposition was up to 74% [4]. It has been reported that intraoperative changes in head position might be one of the causes for tracheal tube shifts [5–7]. In general, in the head flexion position, if the tip of the tracheal tube moves toward the tracheal carina, it may cause endobronchial intubation and single lung ventilation. In contrast, in the maximal head flexion position, if the tracheal tube shifts toward the glottis, tracheal tube prolapse may occur. Moreover, the available formulas are only appropriate for intubation under relatively fixed head-neck positions, mostly the neutral head position. Moreover, to date, there is no specific formula to estimate the intubation depth for pediatric patients when the head-neck position changes during surgery. Therefore, we conducted a prospective study on 172 Chinese children to quantify the impacts of intraoperative head-neck position changes on the depth of oral tracheal tube intubation and attempted to create an appropriate formula for those surgical situations. All included children were in the top 3 percentile of growth.

## Methods

### Participants

This was a prospective study, which included 172 children aged 2–12 years (110 boys and 62 girls) who underwent head and neck surgery with elective general anesthesia in Beijing Tsinghua Chang Gung Hospital from December 2015 to December 2017. For each age group, 13–19 children were included. All children were well-developed, and their height and weight were above the 3rd percentile of the growth curve according to the growth and development study of children in China [8]. Among them, 160 underwent ear, nose, and throat surgery, 7 children underwent orthopedic surgery (facial excision, skin dilator implantation), and 5 children underwent external surgery (intracranial tumor resection). Children who had at least one of the following conditions were excluded: 1) limited head movement, 2) had airway dysplasia (such as airway stenosis, tracheoesophageal fistula), 3) American Society of Anesthesiologists score of III or more.

The study protocol complied with the Helsinki Declaration and was discussed and approved by the ethics committee of Beijing Tsinghua Chang Gung Hospital. The guardian of each child provided signed informed consent.

### Data collection and measurements

We extracted and collected the following general information from the electronic medical records of the patients during the perioperative period: sex, age, height, body weight, surgical type, and adverse effects. Routine records and measurements taken before and during the operation, including vital signs, electrocardiography information, percutaneous oxygen saturation, and noninvasive blood pressure, were also recorded.

Routine general anesthesia was induced in for each patient by slow intravenous injection of sufentanil (0.2–0.3 μg/kg), propofol (2 mg/kg), and rocuronium (0.6 mg/kg). The tracheal tube was inserted according to the depth calculated using the APLS formula—(age/2 + 12) cm—and fixed using tape. Anesthesia was maintained with 1.5–3.0% sevoflurane inhalation (minimum alveolar concentration was maintained between 1.5–2.0), as well as continuous infusion of 2–4 mg/kg/h propofol and 0.1 μg/kg/min of remifentanil using a microinjection pump; sufentanil was injected intermittently. The following breathing parameters were set: tidal volume, 8–10 mL/kg; respiratory rate, 16–26 times/min; end-tidal carbon dioxide, 35–45 mmHg, and intraoperative oxygen concentration, 60%.

**Fig. 1:**
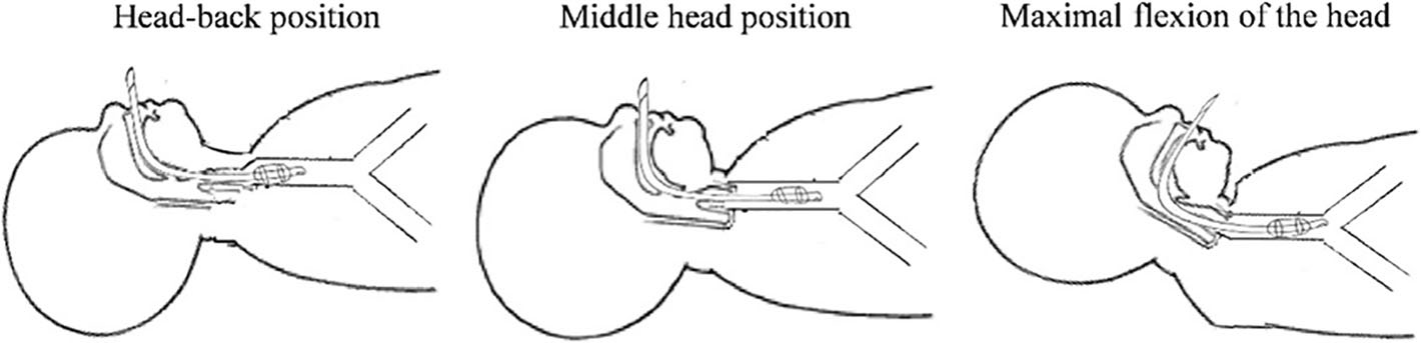
Head position during surgery and the depth of tracheal tube intubation in children

**Fig. 2:**
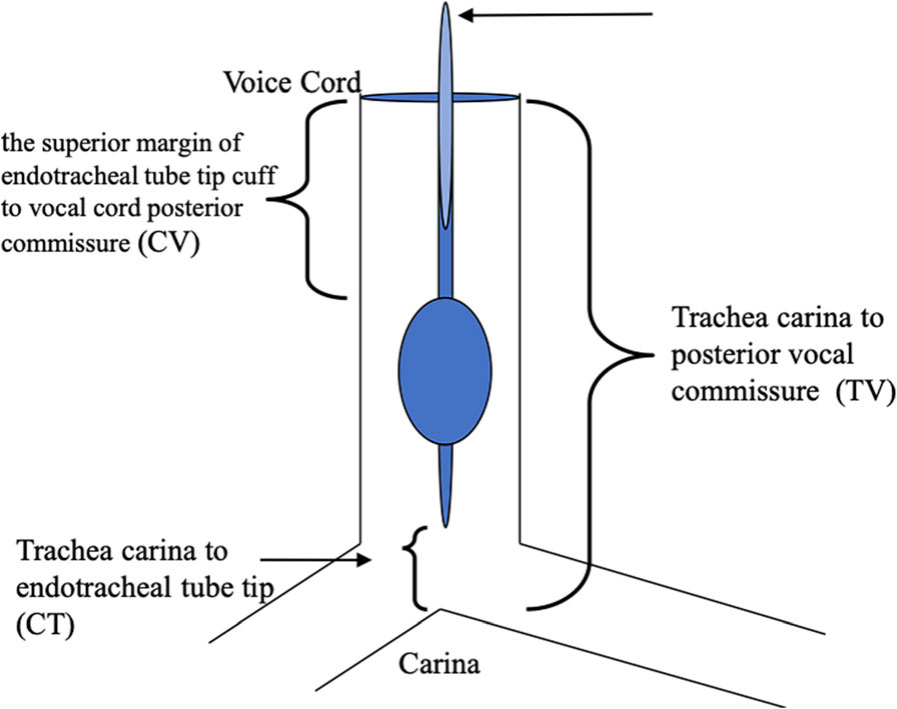
Depth of tracheal tube intubation in children

The children were in the supine position and underwent a fiberoptic bronchoscopy. The distances from the tracheal carina to the endotracheal tube tip (CT), from the superior margin of the endotracheal tube tip cuff to the vocal cord posterior commissure (CV), and between the trachea carina and the posterior vocal commissure (TV or airway length), were measured in the sniffing, median head-neck, and maximum flexion head-neck positions. The surgical head positions are shown in Fig. 1, while the measured distances are shown in Fig. 2.

### Statistical analyses

We calculated the mean ± standard deviation or median (interquartile range) for continuous variables (age, weight, height, BMI, depth of intubation, CT, and TV/airway length) and frequency (percentage) for categorical variables (sex, type of surgery, tracheal prolapse, postoperative hoarseness, and bronchial intubation/single lung ventilation). We compared the differences between the distance (CT, CV, and TV) in the sniffing and maximal head flexion positions with those in the neutral head position using paired t-test. Considering the multiple comparisons, significance was set at *P*-value < 0.025 (Bonferroni correction). Linear regression models were used to fit the estimated models of distance on age. R-square values were calculated to evaluate the goodness of fit.

All analyses were conducted using SPSS 18.0 software (IBM, Chicago). Two-tailed *P*-value < 0.05 was considered statistically significant.

## Results

A total of 172 children were enrolled in the study, including 110 boys and 62 girls (age, 7 years [range, 2–12 years]) (Table 1). Among them, 160 children (93.0%) underwent ENT surgeries (adenotonsillectomy, myringotomy), 7 (4.07%) children underwent orthopedic surgeries (facial nevi excision, skin expander implantation), and 5 (2.91%) children underwent extracranial surgery (craniocerebral tumorectomy). All participants were well-developed children, with a median weight of 24.8 kg (11–84 kg), a median height of 128 cm (87–176 cm), and a mean BMI of 17.17 kg/m^2^ (±3.81 kg/m^2^).

**Table 1:** Characteristics of 172 children included in this study

Characteristics	Total
n	172
Age, year	7 (2–12)
Boys, n (%)	110 (63.95)
Weight, kg	24.8 (11–84)
Height, cm	128 (87–176)
BMI, kg/m^2^	17.17 ± 3.81
Types of surgery, n (%)	
ENT surgery	160 (93.0)
Orthopedic surgery	7 (4.07)
Extracranial surgery	5 (2.91)
Insertion depth (Age/2 + 12 cm), cm	15.5 (14.4, 16.9)
Distance of trachea carina to endotracheal tube tip, cm	4.1 (1.7–9.2)
Distance between trachea carina to posterior vocal commissure, cm	10.5 (7.6–13.5)
Tracheal prolapsing, n (%)	9 (5.23)
Hoarseness after surgery, n (%)	0
Bronchial intubation/single lung ventilation, n (%)	0

### Distances with different intraoperative head and neck positions

In the neutral head position, the mean values of CT, CV, and TV were 4.33 cm ± 1.37 cm, 10.4 cm ± 1.47 cm, and 6.11 cm ± 1.25 cm, respectively. In the sniffing position, CT and CV values increased significantly to 5.43 cm ± 1.46 cm and 11.3 cm ± 1.49 cm, respectively, and TV shortened to 5.90 cm ± 1.20 cm (all *P*-values < 0.025). In contrast, under maximal head flexion position, CT and CV significantly shortened to 3.39 cm ± 1.35 cm and 9.59 cm ± 1.47 cm (all P-values < 0.025), respectively, whereas TV increased slightly to 6.20 cm ± 1.26 cm (*P* = 0.048) (Table 2).

**Table 2:** Measured and calculated distances at 3 surgery positions in 172 children

Distances, cm (mean ± SD)	Head-back position (calculated by APLS formula)	Middle head position	Maximal flexion of the head	P-value for HB vs. MH	P-value for MF vs. MH
CT, cm	5.43 ± 1.46	4.33 ± 1.37	3.39 ± 1.35	< 0.0001	< 0.0001
CV, cm	11.3 ± 1.49	10.4 ± 1.47	9.59 ± 1.47	< 0.0001	< 0.0001
TV, cm	5.90 ± 1.20	6.11 ± 1.25	6.20 ± 1.26	< 0.0001	0.048

**Fig. 3:**
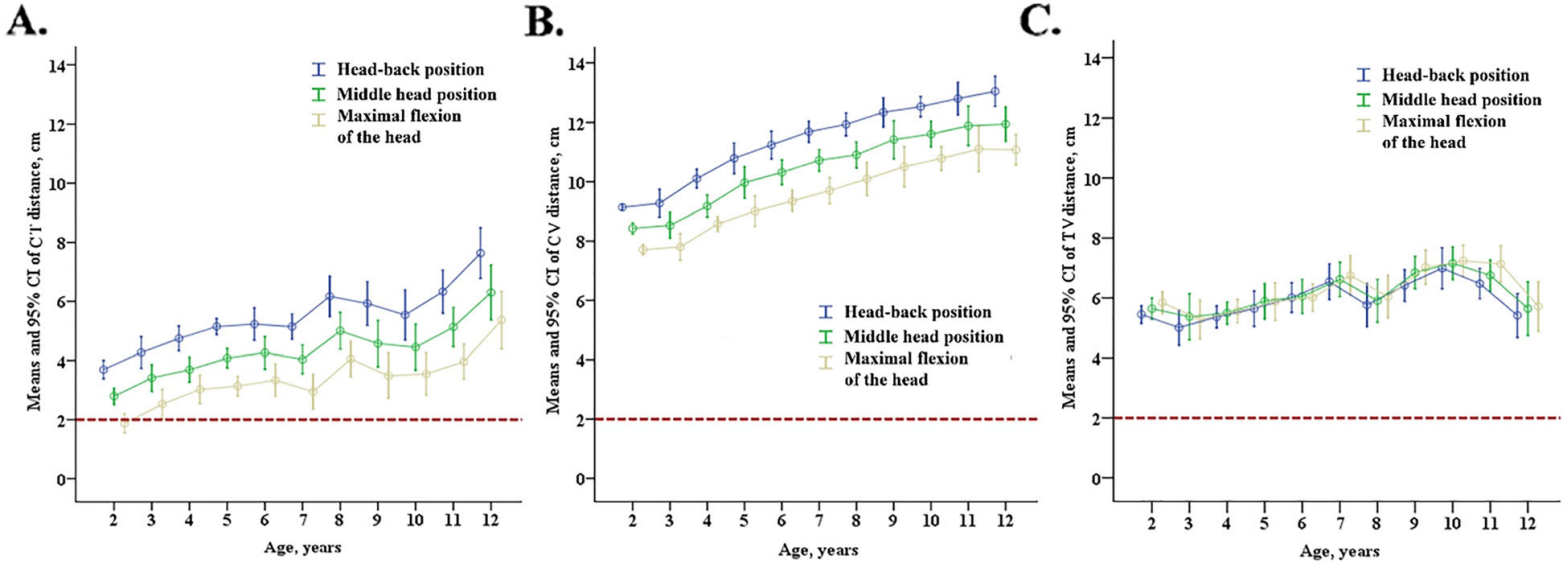
Average distance from the tracheal carina to the endotracheal tube tip (**a**), distance between the tracheal carina to the posterior vocal commissure (**b**), and the distance from the superior margin of the endotracheal tube tip cuff to the vocal cord posterior commissure (**c**) in children with different ages under 3 surgical head positions. The red dotted line indicates that the distance from tip of the tube to bulge is 2 cm, which is the ideal position for the endotracheal tube tip. The error bars represent 95% CIs

On stratification by age (Fig. 3), both CT and CV increased with age. The CT and CV values increased significantly when the head position changed from neutral head to sniffing position, while CT and CV decreased significantly when the head position changed from neutral head to maximal flexion position. The increments and reductions in CT and CV in different age groups were similar.

### Effect of changes in head and neck position on airway length

**Fig. 4:**
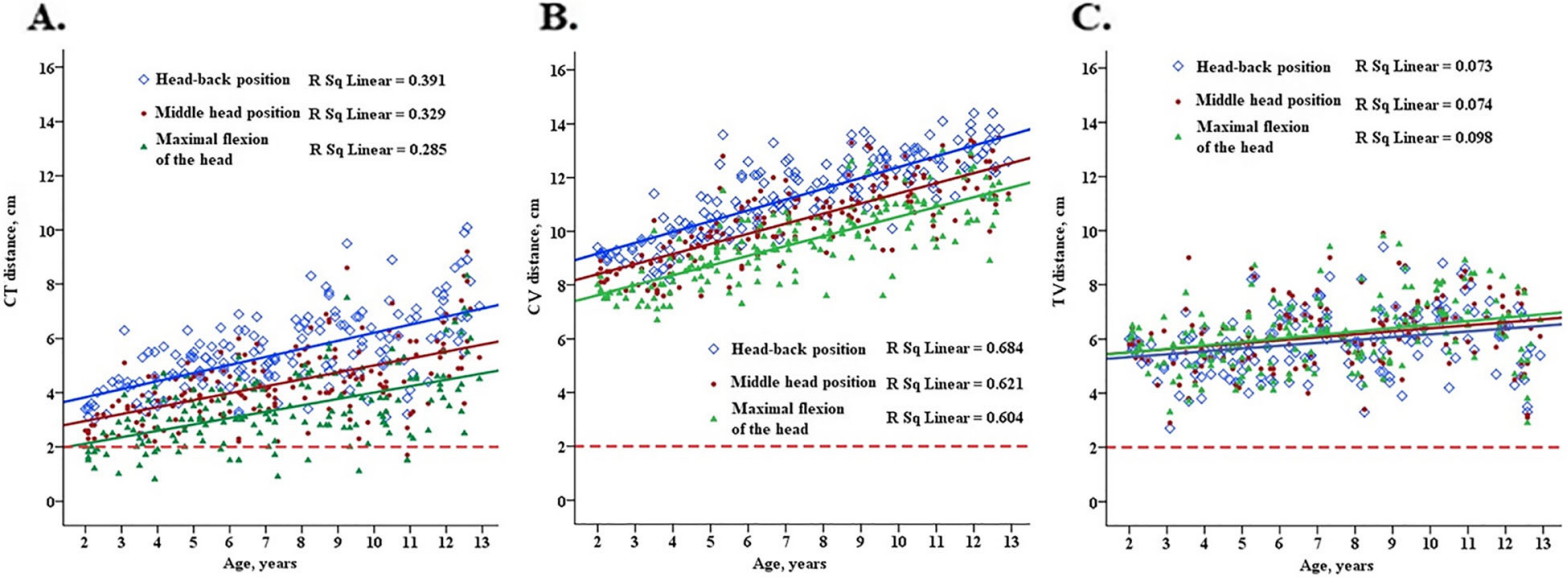
Scatter plot of distance between the tracheal carina to the endotracheal tube tip (**a**), distance from the superior margin of the endotracheal tube tip cuff to the vocal cord posterior commissure (**b**), and the distance between the tracheal carina to the posterior vocal commissure (**c**) with age in children with 3 different intraoperative head positions. The red dotted line indicates that the distance from tip of the tube to the bulge is 2 cm, which is the ideal position for the endotracheal tube tip

Results from our linear regression models suggested that TV did not change with change in head position, but was only dependent on age—TV (cm) = 5 + 0.1 × age. *P*-values for all position changes in the regression models were larger than 0.05, which suggested that the position change might have no effect on TV distance (Table 3; Fig. 4). In contrast, CT and CV changed not only with age but also with the different head positions. When head position was changed from neutral head to sniffing position, both CT and CV increased by 1.099 cm (standard error, 0.122 cm) and 0.909 cm (standard error, 0.094 cm) (all *P*-values < 0.05), respectively. An increment in each year of age was related with an increase of 0.277 cm (standard error, 0.020 cm) of CT and 0.390 cm (standard error, 0.015 cm) of CV. When the head position was changed from middle to maximal head flexion, the reductions in CT and CV were 0.947 cm (standard error, 0.122 cm) and 0.838 cm (standard error, 0.098 cm), respectively (all P-values < 0.05). Moreover, each 1-year increase in age was related with a 0.246-cm (standard error, 0.020 cm) and 0.370-cm (standard error, 0.016 cm) increase in CT and CV values, respectively.

**Table 3:** Linear regression models for 3 distances with age under 3 different surgical positions

Distances, cm	Positions		
	Head-back position	Middle head position	Maximal flexion of the head
CT, cm	CT = 3.236 + 0.298 × age	CT = 2.443 + 0.256 × age	CT = 1.650 + 0.236 × age
CV, cm	CV = 8.362 + 0.403 × age	CV = 7.647 + 0.377 × age	CV = 6.902 + 0.364 × age
TV, cm	TV = 5.125 + 0.105 × age	TV = 5.291 + 0.111 × age	TV = 5.252 + 0.129 × age
		Position changes	
	Middle head position to be Head-back position		Middle head position to be Maximal flexion of the head
CT, cm	CT = 2.290 + 0.277 × age* + 1.099 × Position change*		CT = 2.520 + 0.246 × age*-0.947 × Position change *
CV, cm	CV = 7.550 + 0.390 × age* + 0.909 × Position change *		CV = 7.693 + 0.370 × age*-0.838 × Position change *
TV, cm	TV = 5.312 + 0.108 × age*-0.208 × Position change		TV = 5.226 + 0.120 × age* + 0.90 × Position change

*P-value for regression coefficients < 0.05

### Adverse effects

Tracheal prolapse occurred in 9 children (5.2%), all of whom underwent ENT surgery for adenotonsillectomy and sniffing position. After increasing the intubation depth by 1–2 cm, no tracheal prolapse occurred, and all 9 patients showed good postoperative recovery; no aspiration pneumonia or hoarseness occurred postoperatively. Single lung ventilation due to excessively deep tracheal tube tip position was not observed in any of the 172 children examined.

## Discussion

In this study, we compared the CT, CV, and TV values in 3 common head positions during head and neck surgery in children. We found that CT and CV values changed significantly when the head position shifted from neutral head to sniffing position or maximal flexion. However, TV remained unchanged and was only dependent on age.

Currently, the commonly used formulas for calculating the depth of oro-tracheal intubation in children include the APLS formula, tube diameter formula, tube withdrawal method, and marker method [9, 10]. According to previous reports, the positional suitability rate of the APLS method ranges from 67.9–81% [3, 10, 11], while that reported by another study was only 26% [4]. For the tube diameter formula method, suitability rate ranged between 42 and 76.5%, while it was 73% for the tube withdrawal method [10, 11] and 53% for the catheter marker method [8, 11]. Mariano et al. [10] considered that the tube withdrawal method was more suitable than the formula and marker methods; however, the major complication is the cumbersome operation of the tube withdrawal method. Briefly, the tracheal tube is first inserted into one side of the bronchus; if the breath sound on auscultation is judged to be single lung ventilation, then the tracheal tube is slowly withdrawn. When breath sounds of both lungs are heard on auscultation, the tracheal tube tip is placed on the carina, and the tube is further withdrawn for 2 cm to achieve a suitable depth. Another complication of this method is airway stimulation by the tracheal tube, induction of airway spasm, and airway damage; therefore, it is not a preferred procedure. The easiest intubation method is to place the black marker line of the tracheal tube on the glottis under direct vision. Since the parameter of the catheter from different manufacturers were designed according to the parameters of growth and development of the child. Therefore, whether the depth of the catheter is appropriate dependent on the parameters used, which affects the safety of intubation [12].. However, the data used by tracheal tube manufacturers are mostly derived from European and American children. Because of the ethnic differences on growth and development in children, whether these data are suitable for Chinese children remain unclear.

Our study found that the CT and CV values were dependent on the children’s age and the head-neck position, and that TV remained stable and did not change with changing head positions. Generally, each 1-year increase in age was related with a 0.2-cm, 0.4-cm, and 0.1-cm increase in CT, CV, and TV values, irrespective of the head position. When the head position changed from neutral head to sniffing position, CT and CV values increased by 1 cm; in contrast, the distances decreased by 1 cm when the head position shifted from neutral head to maximal flexion. Our result was similar to that of a previous study [13],which reported that the main airway length increased by 0.95 ± 0.43 cm when the head was at the maximum hypokinesis, and the distance between the endotracheal tube tip and glottis reduced by 1.08 ± 0.47 cm, while the CT increased by 2.02 ± 0.58 cm. All these data indicate that when the head was at a sniffing position, the increased distances for the carina at the tip of the catheter was greater than the increased distance of the airway length. The length of the airway increased, but not proportionate to the movement of the tracheal tube, which caused tracheal tube prolapse after the head position changed.

In this study, 9 children (5.2%) experienced tracheal tube prolapses, all of which were during otolaryngeal surgeries. This surgery required head-neck hypokinesis for a good surgical view. J Lu et al. [14] reported that the incidence of prolapse in children undergoing adenoidectomy was 1.45% (4/276), while Wagner et al. [15] found that prolapse rate was 0.03% during extracranial surgery. During extracranial surgery, the head and neck are fixed, whereas during otolaryngeal surgeries, the surgeon often needs to change the patients’ head position, which was the reason for the high rate of tracheal tube prolapse during this type of surgery. To date, much research has focused on prolapse in children with long-term intubation with a tracheal tube at NICU. Several studies have reported that 3.39–5.3% of children had unplanned extubation [16, 17], and 0.59–0.61% unplanned extubating events/100 intubation days. This evidence suggested that the high-risk factors for unplanned extubation were similar to those of intraoperative prolapse, when tracheal tube was improperly fixed with incomplete patient sedation or lack of operational expertise. This also suggested that for surgeries involving head-neck hypokinesis changes, intubation depth calculated by the APLS formula was shallow, and that the risk of tracheal tube prolapse was higher. The required depth of tracheal tube can be deeper than the original APLS formula.

In medical practice, physicians tend to insert the tracheal tube more deeply than that recommended by the APLS formula [18]. Nicky Lau et al. [5] compared the intubation depth calculated by the classic formula by recording the actual clinical intubation depth in the neutral head position for 137 children aged 1–16 years who underwent oro-tracheal intubation, and considered the following new formula: intubation depth (cm) = age / 2 + 13, which conferred better clinical safety and practicality. However, they did not address the effect of head and neck activity on tracheal tube position. In this study, when a patient’s head was flexed, the tracheal tube could be displaced to the tracheal carina, which may cause endobronchial intubation and single lung ventilation. In fact, we demonstrated that during head flexion, CT shortened by 0.947 (± 0.122) cm. If an extra 1 cm depth was added to the APLS formula, the safety of the airway cannot be guaranteed.

## Conclusions

Our study found that the length of the airway was dependent on children’s age and position of the head and neck. Especially in children undergoing common surgery of the ear, nose, and throat, involving the head and neck hypokinesis, the incidence of tracheal tube prolapse was high. When the head position was changed from neutral head to sniffing position, a 1-cm increase was needed for CT and CV; in contrast, a 1-cm decrease was needed if head position was changed from neutral head to maximal head flexion. Additional well-designed large-scale clinical trials are warrant to confirm our conclusions.

## Data Availability

The datasets used and/or analysed during the current study available from the corresponding author on reasonable request.

## Abbreviations

CT: the distance from the trachea carina to the endotracheal tube tip
CV: the superior margin of the endotracheal tube tip cuff to the vocal cord posterior commissure
TV: the distance from the trachea carina to the posterior vocal commissure
ASA: American Society of Anesthesiologists
SD: standard deviation
